# A bibliometric analysis based on Web of Science from 2012 to 2021: Current situation, hot spots, and global trends of medullary thyroid carcinoma

**DOI:** 10.3389/fonc.2023.1119915

**Published:** 2023-03-07

**Authors:** Ruyin Li, Yingjiao Wang, Zirui Zhao, Xiaobin Li, Ziwen Liu

**Affiliations:** ^1^ Department of General Surgery, Peking Union Medical College, Peking Union Medical College Hospital, Chinese Academy of Medical Sciences, Beijing, China; ^2^ Department of Breast Surgery, Peking Union Medical College Hospital, Peking Union Medical College, Chinese Academy of Medical Sciences, Beijing, China; ^3^ Department of Neurology, Peking Union Medical College Hospital, Peking Union Medical College, Chinese Academy of Medical Sciences, Beijing, China

**Keywords:** medullary thyroid carcinoma, Web of Science, VOSviewer, trends, bibliometric analysis

## Abstract

**Background:**

Medullary thyroid carcinoma (MTC) is a special type of thyroid carcinoma derived from the C cell of the thyroid gland. Because of the poor prognosis of MTC, a large number of studies on MTC have been conducted in the last 10 years. To better comprehend, it is necessary to clarify and define the dominant countries, organizations, core journals, important authors, and their cumulative research contributions, as well as the cooperative relationships between them.

**Method:**

English publications with article type article or review about MTC from January 2012 to December 2021 was retrieved from Web of Science core collection, and VOSviewer, CiteSpace, and Microsoft Excel were applied for bibliometric study.

**Result:**

A total of 1208 articles and reviews were included in this study. The 1208 papers were written by 6364 authors from 1734 organizations in 67 countries, published in 408 journals, and cited 24118 references from 3562 journals. The number of publications was essentially flat from 2012-2021, with the largest proportion of publications coming from the U.S., followed by Italy and China. Thyroid was the most productive journal, and Journal of clinical endocrinology & metabolism was the most cited journal. University of Texas MD Anderson Cancer Center was the most productive institution and Luca Giovanella, was the most productive author. Diagnostic tools, surgical treatment, non-surgical treatment, genetics and relationship with other endocrine diseases were the main research interests in this field. Prognosis has been a cutting-edge topic since 2017.

**Conclusion:**

As a thyroid cancer with poor prognosis, MTC has received continuous attention in recent years. Current MTC studies mainly focused on disease intervention, mechanism research and prognosis. The main point of this study is to provide an overview of the development process and hot spots of MTC in the last decade. These might provide ideas for further research in the MTC field.

## Introduction

Thyroid cancer is the most common endocrine malignant tumor. According to the histopathological type, it can be divided into medullary thyroid carcinoma (MTC), anaplastic thyroid carcinoma and differentiated thyroid cancer(DTC), which includes papillary thyroid cancer, follicular thyroid cancer (1). MTC is a special type of thyroid cancer derived from the C cell of the thyroid gland, with elevated plasma calcitonin and carcinoembryonic antigen, accounting for about 2% of all thyroid carcinomas (2). Depending on the clinical symptoms of MTC, it can be classified as hereditary (75%) and sporadic (25%) (3). Compared with DTC, MTC is more likely to find local lymph nodes metastasis, and the overall 10-year survival rate of MTC ranges 61-76% (4). Total thyroidectomy and dissection with or without cervical lymph node compartments is standard treatment for patients with MTC (5). Recently, as a poorly prognosed thyroid carcinoma, a large number of studies have been conducted on MTC.

Bibliometric analysis is an effective tool. By summarizing and analyzing the data of countries and regions, journals, authors, keywords, citations, bibliometric analysis can visually reveal the developing process, current status and hotspots of researches in one field as well as predict the global trends in the future (6, 7).

Recently, the application of bibliometric analysis in the field of thyroid research is focused on diseases such as papillary thyroid cancer (8) and anaplastic thyroid cancer (9). To our acknowledge, there is no bibliometric analysis on MTC. Then, we searched “medullary thyroid carcinoma OR thyroid AND carcinoma, medullary” and “Bibliometrics OR Bibliometric” on Web of Science and PubMed, and no relevant papers were found. As the first bibliometric analysis on MTC, this study aims to use bibliometric methods to reveal the development process, current status, hotspots and predict global future trends in the field of MTC research.

## Materials and methods

### Data source

Web Of Science (https://access.clarivate.com) is a database of high-quality articles covering multiple fields, most commonly used for bibliometric analysis (10). Compared to PubMed and Scopus, Web of Science has a greater reach, higher quality literature, and easier access to citation information for articles (11). In order to make the target article information more accurate and comprehensive, this research chose Web of Science Core Collection as data source and set the citation indexes as Science Citation Index Expanded and Social Sciences Citation Index.

Further, since the corresponding search strategy for MTC in the Medical Subject Headings Database (MeSH) is “Thyroid AND Carcinoma, medullary”, we determined the subject term search formula as “TS=Thyroid AND Carcinoma, medullary “, and set the time span from January 1, 2012 to December 31, 2021, the article type as “Article” or “Review”, and the language as “English”. Ultimately, the full search formula for obtaining data is as follows.((TS=(Thyroid AND Carcinoma, medullary)) AND LA=(English)) AND DT=(Article OR Review). (This search was completed on November 21, 2022).

Finally, a total of 2056 articles were retrieved. After browsing article abstracts, we removed 851 articles with large deviations from the topic and 1 duplication, resulting in a total of 1208 valid articles, including 947articles and 265 reviews. The 1208 papers used in this study were authored by 6364 authors from 1734 organizations in 67 countries. And they were published in 408 journals and 24118 references were cited from 3562 journals.

### Method

VOSviewer and CitesSpace are bibliometric softwares and both pay special attention to the graphical representation of bibliometric maps. VOSviewer applies probabilistic-based data normalization methods to produce bibliometric maps, including network visualization, overlay visualization and density visualization. The main function of VOSviewer is to cluster data and present co-occurrence relationships between data, usually used to analyze authors, countries, journals, citations, and keywords. The significant advantage of VOSviewer is that the map is beautiful and easy to understand (12). CiteSpace applies a set theory-based data normalization approach to produce bibliometric maps. The main functions of CiteSpace and VOSviewer are basically the same. However CiteSpace is often used to analyze keywords. The advantage of CiteSpace is that a timeline view of the data (Timeline) and the burst words can be obtained, which allows a clear presentation of the research development process and frontiers in a field (13).

**Figure 1:**
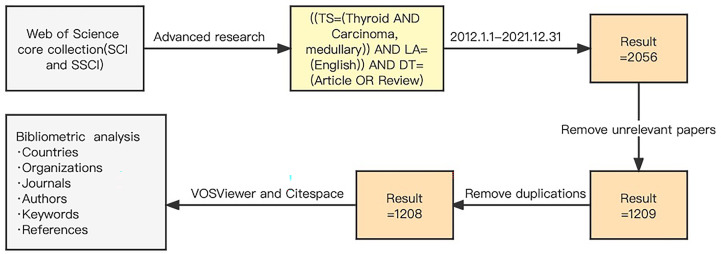
Search strategy, data screening and bibliometric analysis steps.

In this study, VOSviewer (Version 1.6.18), CitesSpace (Version 6.1.R4), and Microsoft Excel were applied to analyze and map the distribution of publications, countries, institutions, authors, journals, keywords and citations of the retrieved 1208 articles. The search strategy, data screening and bibliometric analysis steps can be found in **Figure 1**.

## Results

### Distribution of publications by year

**Figure 2:**
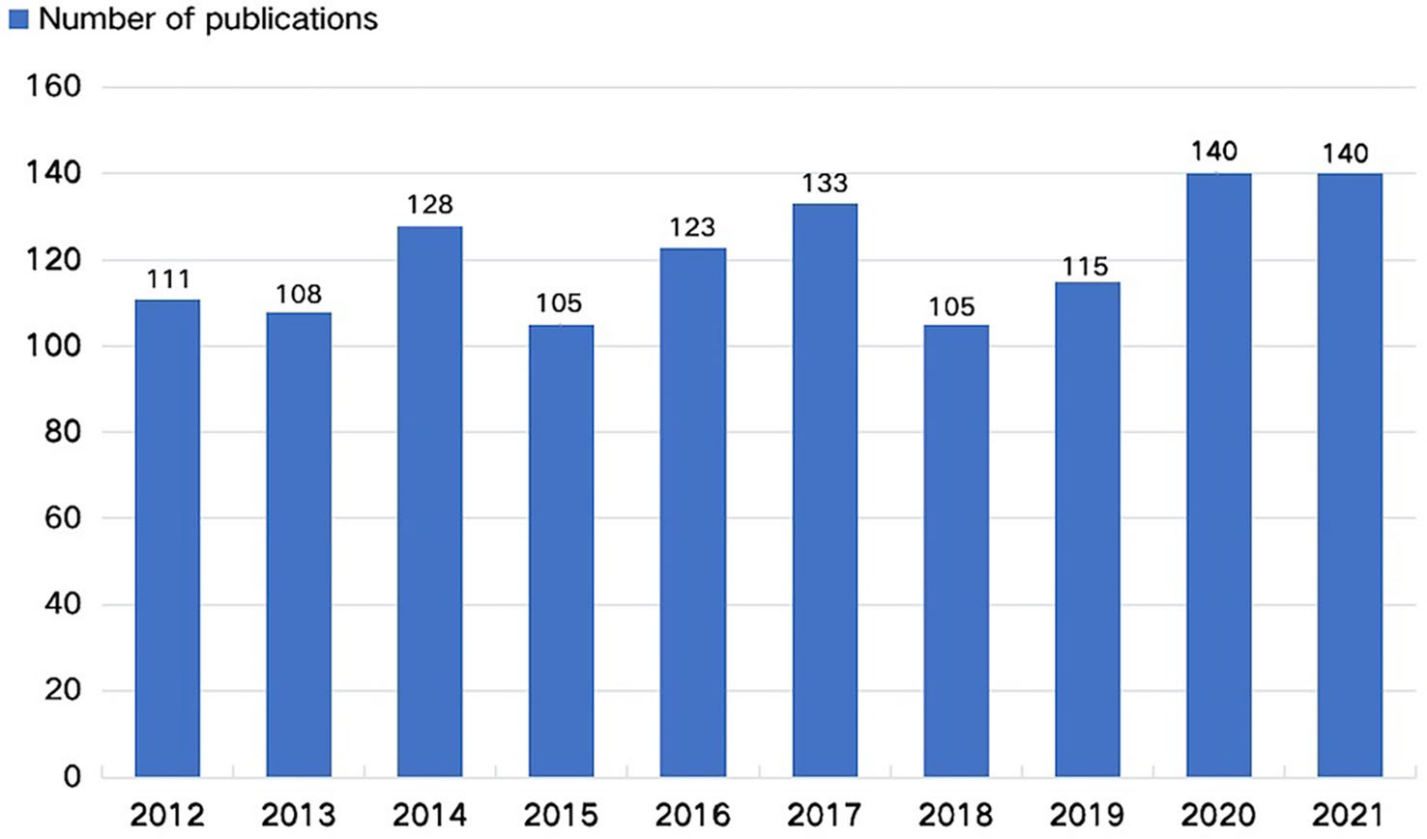
Time trend of the publications on MTC.

To better understand the current status of publications in the field of MTC, this study analyzed the distribution of publications by year from 2012 to 2021.Time trend of the publications on MTC research can be seen in **Figure 2**. The annual number of publications in the field of MTC had been stable at 105 and above in the past 10 years. Annual publication volume peaked at 140 articles in 2020 and 2021.

### Countries

**Figure 3:**
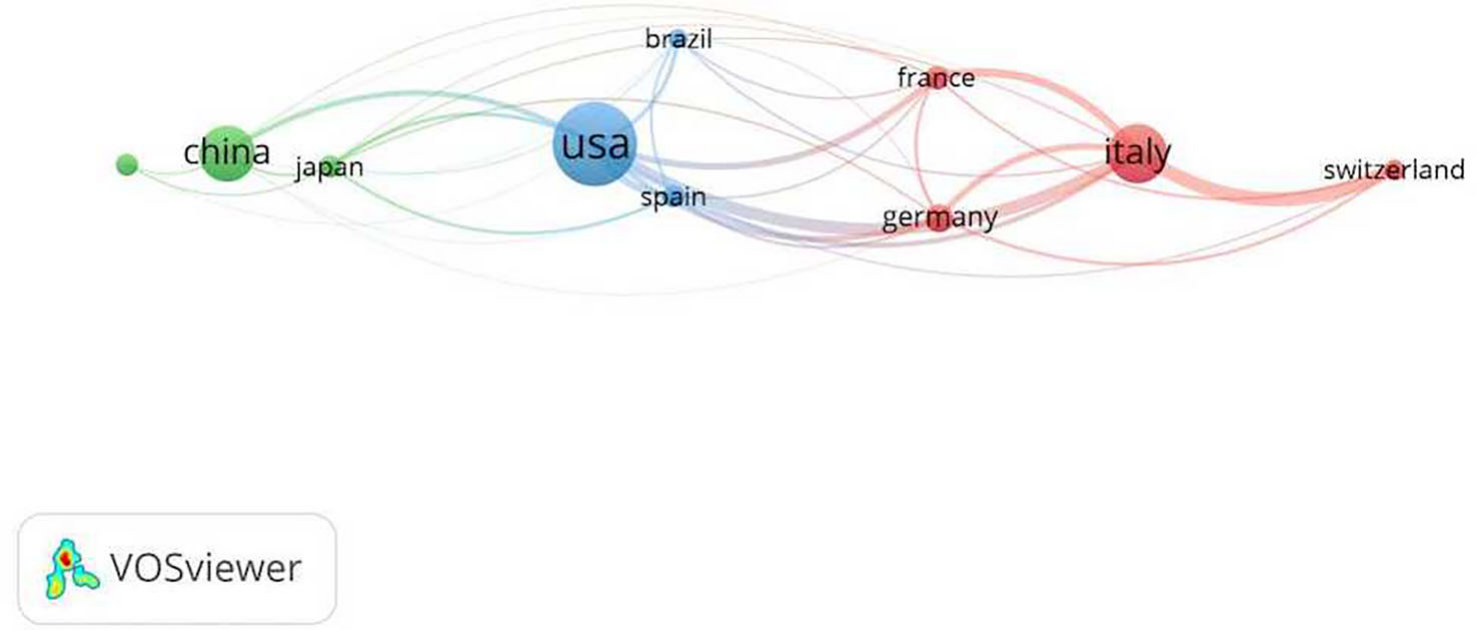
Co-occurrence of the 10 most productive countries.

To clarify the contribution of different countries to research on MTC, this study conducted an bibliometric analysis of the 67 countries involved. Setting the minimum number of national publications at 42 through VOSViewer, we obtained the top 10 countries in terms of the number of publications (**Table 1**) and visualized the cooperation between the top 10 countries (**Figure 3**).

**Table 1:** Top 10 countries in the MTC research field.

Rank	Country	Publications	Citations	Average Citation/Publication
1	Usa	299	7163	23.96
2	Italy	188	4593	24.43
3	China	172	1652	9.60
4	Germany	69	2441	35.38
5	France	56	2090	37.32
6	Spain	52	735	14.13
7	Japan	49	585	11.94
8	South korea	49	556	11.35
9	Switzerland	45	996	22.13
10	Brazil	43	537	12.49

**Table 1** showed that the top three productive countries were the United States, Italy and China. The top cited countries were the United States, Italy and Germany. And the three countries with the highest average citation rate were France, Germany and Italy.

In **Figure 3**, larger nodes suggested more postings, and thicker lines between two countries suggested more cooperation. From this picture, we got the following information:1. The three countries that cooperated the most with other countries were Italy, the United States and China.2. Italy mainly cooperated with European countries, while the United States cooperated with all countries, and China cooperated with all countries except Switzerland. 3. Italy and the United States had much more international cooperation than China.

### Organizations

According to VOSviewer analysis, 1734 different organizations were involved in the research worldwide. Setting the minimum number of articles issued by the institution at 17, the top 10 organizations ranked by document numbers were shown in **Table 2** with total citations and countries. As could be seen from **Table 2**, the top three institutions in terms of number of publications were University of Texas MD Anderson Cancer Center, University of Pisa, University of Padua. University of Texas MD Anderson Cancer Center had the highest number of citations. Moreover, four of the 10 most productive institutions were from Italy.

**Table 2:** Top 10 organizations in the MTC research field.

Rank	Organizations	Publications	Citations	Country
1	University of Texas MD Anderson Cancer Center	38	2273	USA
2	University of Pisa	35	1830	Italy
3	University of Padua	26	375	Italy
4	University of Naples Federico II	20	1607	Italy
5	Sapienza University of Rome	20	606	Italy
6	Memorial Sloan-Kettering Cancer Center	19	1465	USA
7	Zhejiang university	18	257	China
8	KUMA hospital	17	235	Japan
9	Oncology Institute of Southern Switzerland	17	471	Switzerland
10	Radboud University Nijmegen	17	355	Netherlands

### Authors

A total of 6364 authors were further analyzed using the VOSViewer. The top ten productive authors could be obtained by setting the minimum number of publications to 11(**Table 3**). Among these productive authors, the most productive was Luca Giovanella from New Zealand. From January 1, 2012 to December 31, 20221, he had published 24 publications with 575 citations and average citations of 23.96.The second most published and most cited author was Rossella Elisei from the University of Pisa, Italy. From January 1, 2012 to December 31, 20221, he had published 18 publications with 1,351 citations and average citations of 75.06. The two authors with the most average citation were Henning Dralle and Andreas Machens. They were from the same institution and had co-authored 13 articles with 1315 citations and average citations of 100.46.

**Table 3:** The most important authors in the MTC research field.

Rank	Author	Documents	Citations	Average Citation/Publication
1	Luca Giovanella	24	575	23.96
2	Rossella Elisei	18	1351	75.06
3	Pierpaolo Trimboli	16	473	29.56
4	Alessandro Antonelli	14	334	23.86
5	Henning Dralle	13	1315	100.46
6	Andreas Machens	13	1315	100.46
7	Cristina Romei	13	221	17
8	Poupak Fallahi	12	284	23.67
9	Caterina Mian	12	186	15.5
10	Barbara Jarząb	11	126	11.45

### Journals

Bradford proposed that the ratio of core, related and peripheral journals in a field for a given time period is 1:*n*:*n* ^2^ (14). There were 408 journals involved in this study, so the number of the core journal was about 8. The top 8 productive journals could be obtained by setting the minimum number of publications to 18 through VOSviewer(**Table 4**). And the main directions of these journals were cancers and diseases derived from the thyroid, endocrine system, head and neck. Thyroid, Endocrine, and Diagnostic cytopathology were the top three journals with the highest publication. The journal with the most average citations was Journal of clinical endocrinology & metabolism, which published 19 MTC-related articles with 1,029 citations and 54.16 average citations. It was followed by Thyroid with 41 publications and 1,889 citations, with an average citation of 46.07.

**Table 4:** Top 8 journals in the MTC research field.

Rank	Source	Publications	Citations	Average Citation/Publication
1	Thyroid	41	1889	46.07
2	Endocrine	33	355	10.76
3	Diagnostic cytopathology	31	299	9.65
4	Endocrine-related cancer	30	771	25.7
5	Endocrine pathology	29	316	10.90
6	Frontiers in endocrinology	19	143	7.53
7	Journal of clinical endocrinology & metabolism	19	1029	54.16
8	Head and neck-journal for the sciences and specialties of the head and neck	18	229	12.72

### Keywords

**Figure 4:**
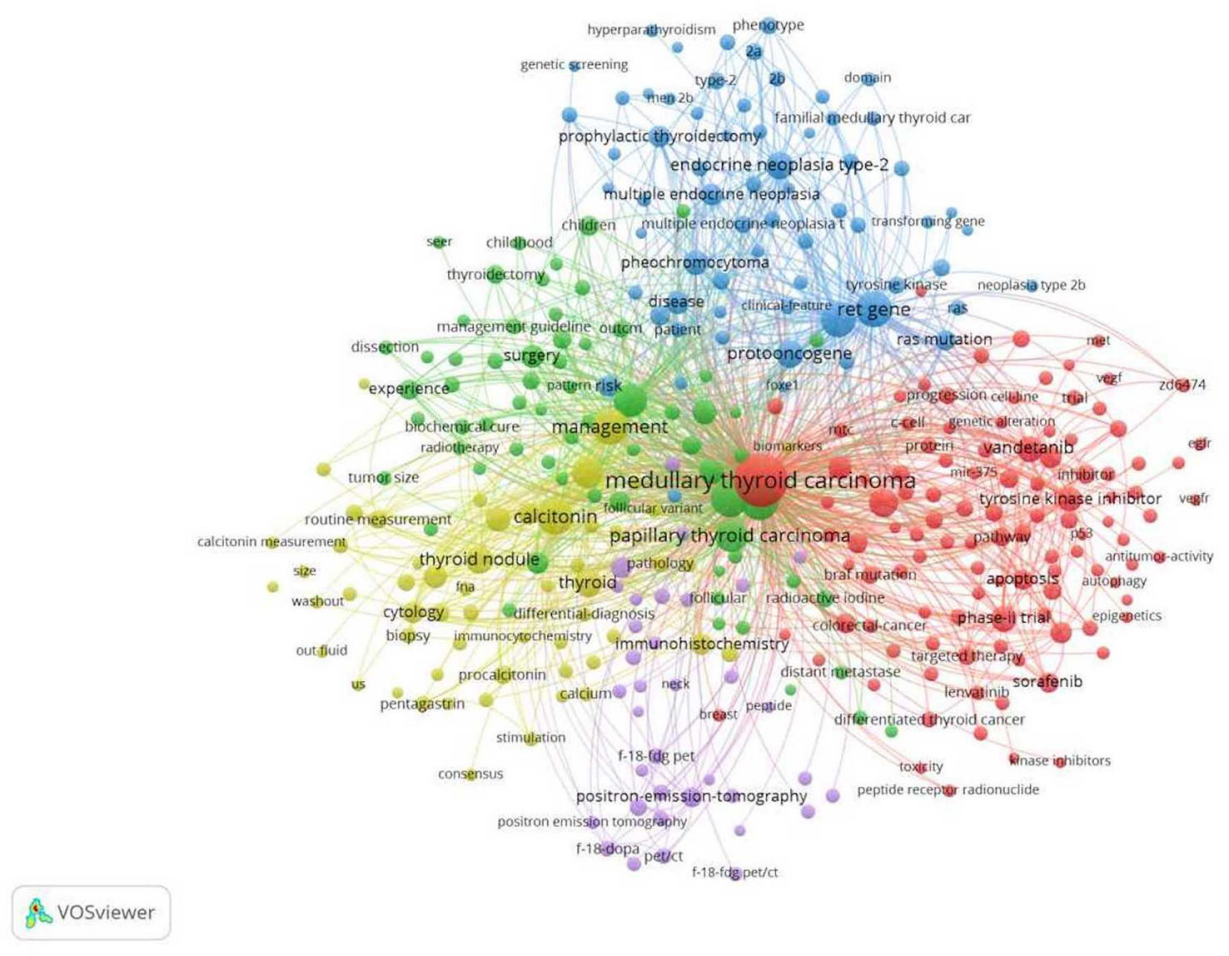
Co-occurrence of the keywords.

A total of 3964 keywords were extracted from 1208 articles. The relationship between the 300 most frequent keywords were visualized by VOSViewer as follows (**Figure 4**). According to **Figure 4**, we found that all the keywords centered around MTC. These keywords were divided into 4 main clusters: non-surgical treatment (red cluster), surgical treatment (green cluster), genetic changes and correlation with other endocrine diseases (blue cluster), diagnosis (yellow and purple clusters), which included nodal ultrasound and biopsy, and also PET/CT for MTC. The minimum number of occurrences of a key word was set to 64 in VOSViewer, and the top 20 keywords were selected and listed in **Table 5**. The top three keywords were medullary thyroid carcinoma, carcinoma, and prognosis.

**Table 5:** The top 20 keywords in the MTC research field.

Rank	Source	Publications
1	medullary thyroid carcinoma	1001
2	carcinoma	326
3	prognosis	273
4	ret gene	268
5	management	267
6	calcitonin	242
7	gene mutation	229
8	guideline	178
9	diagnosis	163
10	papillary thyroid carcinoma	157
11	expression	109
12	endocrine neoplasia type-2	104
13	thyroid nodule	95
14	protooncogene	94
15	thyroid	90
16	vandetanib	81
17	phase-ii trial	74
18	pheochromocytoma	70
19	tyrosine kinase inhibitor	65
20	fine-needle-aspiration	64

**Figure 5:**
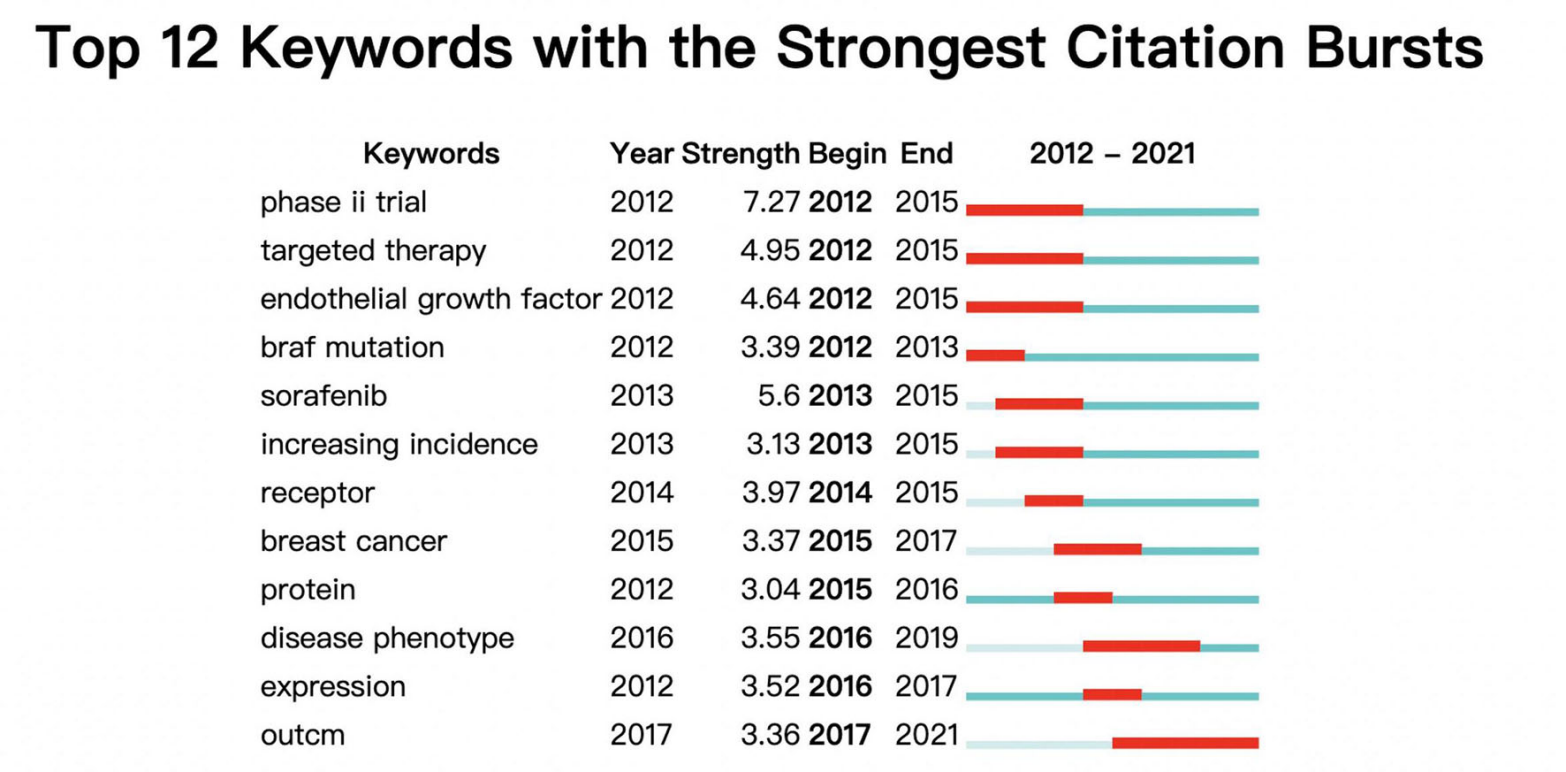
The top 12 burst words.

Burst words can reflect the hot spots of research in a field at different times. Citespace was applied to analyze 3964 keywords contained in 1208 articles for burst words, and a total of 43 burst words were filtered. Excluding the 27 burst words with strength less than 3 and 4 non-informative terms, a total of 12 valid burst words were captured (**Figure 5**). In the early period from 2012 to 2021, hot spots in MTC research included phase II clinical trials (began in 2012 ended in 2015), targeted therapies (began in 2012 ended in 2015), epithelial growth factor (began in 2012 ended in 2015), *BRAF* mutations (began in 2012 ended in 2013), while later research hot spots included sorafenib(began in 2013ended in 2015),increasing incidence (began in 2013 ended in 2015), receptor (began in 2014 ended in 2015), breast cancer (began in 2015 ended in 2017), protein (began in 2015 ended in 2016), disease phenotype (began in 2016 ended in 2019), expression (began in 2016 ended in 2017). Since 2017, the burst word outcome has become a new research hotspot.

### Citations

**Figure 6:**
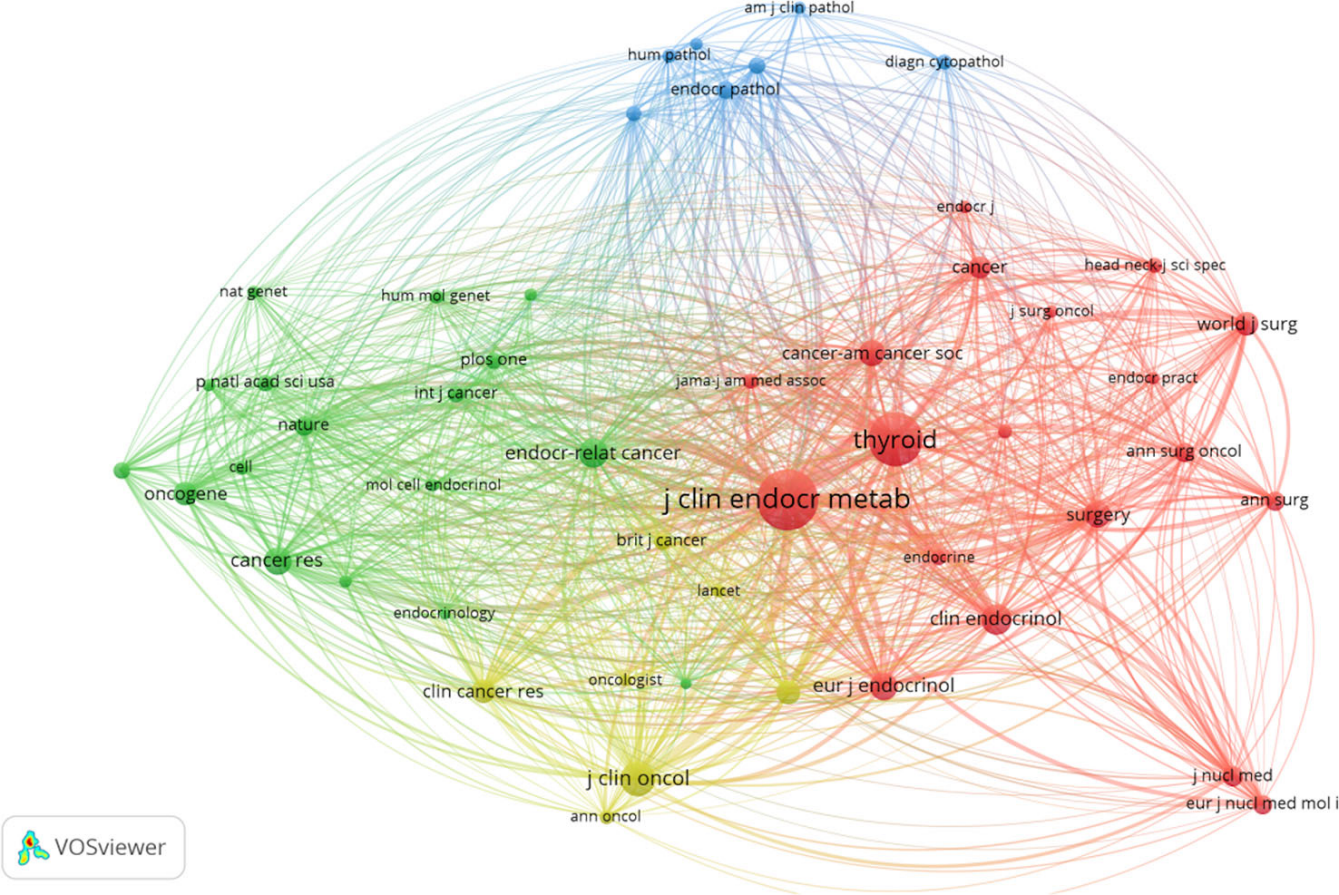
Co-occurrence of the top 50 cited journals.

VOSViewer was used to filter the top 10 cited articles among 1208 articles, and the minimum number of citations was set to 100 to get **Table 6**.The majority of the 10 highly cited articles were review articles, mostly on guidelines and targeted therapies for MTC. Applying VOSviewer to filter 24118 citations from 3562 journals and setting the mini mum number of citations to 160, the top 50 cited journals were obtained and their co-citation relationships were shown in **Figure 6**.It revealed a strong collaboration between journals in the field of MTC research, and the top three cited journals were Journal of clinical endocrinology & metabolism, Thyroid, and Journal of clinical oncology. The co-citation network of the journal included four clusters, including endocrine-related and surgery (red cluster), pathology (blue cluster), and oncology, molecular direction (green and yellow clusters).

**Table 6:** Top 10 highly cited documents in the MTC research field.

Rank	Tittle	Author	Journal	Citations	Year
1	Revised American Thyroid Association Guidelines for the Management of Medullary Thyroid Carcinoma	Samuel A. WellsJr	thyroid	1033	2015
2	Targeted Next-Generation Sequencing Panel (ThyroSeq) for Detection of Mutations in Thyroid Cancer	Marina N.Nikiforova	The Journal of Clinical Endocrinology & Metabolism	316	2013
3	Multiple Endocrine Neoplasia Type 2 and Familial Medullary Thyroid Carcinoma: An Update	Samuel A.WellsJr	*The Journal of Clinical Endocrinology & Metabolism*	188	2013
4	German Association of Endocrine Surgeons practice guideline for the surgical management of malignant thyroid tumors	Henning Dralle	Langenbeck’s Archives of Surgery	144	2013
5	NCCN Guidelines Insights: Thyroid Carcinoma, Version 2.2018	Robert I Haddad	Journal of the National Comprehensive Cancer Network: JNCCN	136	2018
6	Thyroid carcinoma, version 2.2014	R Michael Tuttle	Journal of the National Comprehensive Cancer Network: JNCCN	114	2014
7	The Role of Cdk5 in Neuroendocrine Thyroid Cancer	Karine Pozo	Cancer Cell	112	2016
8	Overall survival analysis of EXAM, a phase III trial of cabozantinib in patients with radiographically progressive medullary thyroid carcinoma	M. Schlumberger	Annals of oncology: official journal of the European Society for Medical Oncology	106	2017
9	Targeted Therapy for Advanced Thyroid Cancer: Kinase Inhibitors and Beyond	Maria E Cabanillas	Endocrine reviews	104	2019
10	Lenvatinib: Role in thyroid cancer and other solid tumors	Maria E Cabanillas	Cancer treatment reviews	104	2016

## Discussion

MTC is a rare type of thyroid cancer with a poor prognosis. In recent years, researchers have conducted numerous studies on the pathogenesis and treatment of MTC in order to improve the prognosis of MTC patients, while no bibliometric article on MTC has been published worldwide. This paper is the first bibliometric analysis on the field of MTC research, visualizing 1208 publications in the field of MTC from 2012 to 2021 in terms of trends in publication, most scientifically productive countries, organizations, journals, authors, keywords and citations. This research reveals the development process, current research status and hotspots in the field of MTC from 2012 to 2021, and predicts global trends in this field.

From 2012 to 2021, the average annual number of articles in the MTC field was stable above 105, which reached a peak of 140 articles per year in 2020 and 2021 (**Figure 2**), indicating that MTC had been a hot pot and received increasing attention in recent years.

The top ten most productive countries were concentrated in the Americas, Asia and Europe, which proved that the publication situation in this research area was not evenly distributed at the world level. And the United States and Italy were leading in publication energy and citations, further revealing the dominance of the United States and Italy in the field of MTC research and the high quality of publications (**Table 1**). The partnership mapping of the top ten countries of publication showed that the United States, Italy, and China, which had the highest number of publications, had more cooperation with other countries, suggesting frequent cooperation among the most productive countries in MTC research and a good international exchange pattern (**Figure 3**).

An analysis of the organization of the literature is key to identifying the core organizations in the field. The top three publishers among the 1,734 institutions covered in this article were University of Texas MD Anderson Cancer Center, University of Pisa, and University of Padua, all from the United States or Italy. In addition, the top ten organizations publishing papers were from the United States and Italy, accounting for six of them. This result was consistent with the results of the national bibliometric analysis, proving once again that the United States and Italy were located at the center of this research field (**Table 2**).

Analysis of the authors of the papers reveals the representative scholars and core research strengths in the field of research. The top author in the field of MTC in the last 10 years was Luca Giovanella, with 24 publications, mainly devoted to the study of MTC diagnosis (15), including the evaluation of the diagnostic efficacy of fine needle aspiration cytology (16) and calcitonin levels in fine needle aspiration cytology (17), ultrasound (2, 18) and positron emission tomography/computed tomography (19). The second most published and most cited author, Rossella Elisei, whose main research interests were targeted therapies (20) and *RET* mutations (21). He was engaged in the revision of the 2015 edition of the MTC management guideline, which was responsible for its most cited authorship. His engagement in the revision of the 2015 edition of the MTC management guideline was what made him the most cited author. With the most average citations, Henning Dralle and Andreas Machens came from the same institution, and were also engaged in the revision of the 2015 edition of the MTC management guideline, their main research interests were the prognosis of MTC surgery at different stages (22, 23). All of these authors were leading scholars in the field of MTC research and had played an important role in the development of the field.

According to Bradford’s law, we calculated the number of core journals in the field of MTC from 2012 to 2021 to be about 8 (**Table 4**), and the 3 most published journals were Thyroid, Endocrine, and Diagnostic cytopathology, with the most cited journal being Journal of clinical endocrinology & metabolism. These journals were mostly in the field of thyroid and internal division diseases and their pathology, all of which were of great importance and influence in the field of thyroid.

The keyword co-occurrence map reflects the distribution of research directions in a given field over time. **Figure 4** revealed that the main research directions in the field of MTC research in the last 10 years had been diagnostic, non-surgical treatment, surgical treatment, genetic and related to other endocrine diseases. The table of the top 20 most important keywords (**Table 5**) indicated that prognosis of MTC, *RET* gene mutations, therapeutic measures, plasma calcitonin, and interventions had been the main research features in the field of MTC in the last 10 years. Burst keywords represent the cutting-edge topics in a certain field at a certain time. The 12 burst words in **Figure 5** indicated that between 2012 and 2021, the research frontier in this field had gone through a total of three phases. The first phase was dominated by targeted therapies and clinical trials, the second phase, by molecular research, and the third phase, by outcome. Meanwhile outcome is also a recent research frontier in the field of MTC and a global trend in the coming years.

Analyzing highly cited literature helps to have a clearer understanding of the current development of a field. As shown in **Table 6**, articles with article type review, content MTC guidelines, and targeted therapies were prone to be highly cited articles in this field. The 2015 edition of the revised MTC management guideline (5) states that FNAC, ultrasound, plasma calcitonin concentration, carcinoembryonic antigen values, and *RET* gene mutation status can be used as assists in the diagnosis of MTC. For patients without distant metastases, total thyroidectomy and dissection with or without cervical lymph nodes metastasis is the primary recommended therapy. For patients with MTC with distant metastases, treatment of the original site or systemic therapy, such as systemic therapy with tyrosine kinase inhibitors and clinical trial, can be considered. tyrosine kinase inhibitors is the most commonly used targeted therapy with MTC. In recent years, vandetanib and cabozantinib have been used to treat advanced MTC, and studies of their efficacy are gradually being conducted (24–26). At the same time, the efficacy of other tyrosine kinase inhibitors drugs such as sunitinib in MTC is also being investigated (27, 28).

Co-citation analysis can characterize the internal structure of knowledge based on co-cited references. **Figure 6** showed the top 50 cited journals among 1208 articles. These journals could be roughly divided into 4 major categories, covering endocrine-related and surgical direction, MTC pathology direction, MTC oncology, molecular. Citing these articles provided a basis for clinical interventions, facilitates a more in-depth understanding of the pathological features of MTC, and provided a basis or ideas for the exploration of tumor mechanisms.

This study also has some limitations. Firstly, these articles are from the Web of Science core collection of Science Citation Index Expanded and Social Sciences Citation Index. Secondly, the language of the publications is set to English and the type of the publication is set to article or review. These mentioned above may make some relevant publications missing. However, it is generally accepted that these articles represent a very small fraction of all articles and have little impact on the result of our findings (29). The inclusion of case reports and cases series in the bibliometric analysis could be considered in future studies, which may provide more precise hints on the direction of research at the frontiers of MTC, such as rare gene fusions and TKI therapeutic breakthroughs.

In general, the research of MTC is currently in a booming phase. Gene mutation, targeted therapy, mechanism and prognosis of MTC are all hot directions, and, outcome is the frontier hot spot. In the future, more related research is expected to be conducted, and research based on the above directions will become the mainstream of MTC research, which is important for the diagnosis and treatment of MTC and improving the prognosis of patients.

## Conclusion

In summary, researchers had conducted numerous studies on MTC from 2012 to 2021.The United States and Italy and their institutions were leaders in MTC research, with the highest scientific productivity. Luca Giovanella was the most published author. Thyroid, Endocrine, Diagnostic cytopathology, Journal of clinical endocrinology & metabolism were the most important core journals in the field. Outcome, *RET* gene mutations, interventions, plasma calcitonin, and targeted therapy were hot research topics in the field of MTC. Outcome was a cutting-edge topic and global trend in the field of MTC research.

Overall, this study is the first bibliometric analysis of MTC research. This study analyses the publications in the field of MTC and presents them in a visual way, revealing to a certain extent the development process, research hotspots and future global trends, which may provide ideas for further research.

## Data availability statement

The raw data supporting the conclusions of this article will be made available by the authors, without undue reservation.

## Author contributions

This work was conceived by RL. Data was collected and downloaded by RL, WY and ZZ. The visualization work was performed by RL. The manuscript was written by RL. XL, ZL, WY and ZZ helped to revise manuscript and proposed constructive opinions. All authors contributed to the article and approved the submitted version.
